# Septic Arthritis Associated With Hip Joint Subluxation and Epiphyseal Plate Deformation as a Sequala of Sickle Cell Anemia

**DOI:** 10.7759/cureus.48103

**Published:** 2023-11-01

**Authors:** Harsh R Nathani, Subrat Samal

**Affiliations:** 1 Department of Musculoskeletal Physiotherapy, Ravi Nair Physiotherapy College, Datta Meghe Institute of Higher Education and Research, Wardha, IND

**Keywords:** comprehensive rehabilitation, sickle cell anemia, hip subluxation, pediatric patient, septic arthritis

## Abstract

This case report emphasizes the critical nature of septic arthritis in pediatric patients, particularly its predilection for large joints like the hip. It underscores the importance of rapid diagnosis and early intervention to preserve joint function. The case involves a six-year-old patient with sickle cell anemia who presented with persistent hip pain and hip subluxation and underwent hip joint arthrotomy. Clinical findings revealed limited mobility, weakness, and radiographic abnormalities. Comprehensive rehabilitation resulted in significant improvements in pain, mobility, and function. The key takeaway is the pivotal role of early diagnosis and comprehensive rehabilitation in managing septic arthritis in pediatric patients, especially those with underlying conditions like sickle cell anemia.

## Introduction

Septic arthritis denotes the inflammatory response affecting a joint, precipitated by an infectious etiology, most frequently bacterial, with occasional instances linked to fungal, mycobacterial, viral, or less common microbial agents. Typically, septic arthritis manifests as a monoarticular condition, afflicting a solitary, sizable articulation, such as the coxofemoral or tibiofemoral. Nevertheless, infrequently, it may present as septic arthropathy involving multiple joints, affecting numerous or diminutive joints. Despite its infrequency, septic arthritis constitutes a musculoskeletal medical urgency that bears the potential for substantial joint impairment, leading to heightened levels of morbidity and mortality. Timely diagnosis and intervention stand as critical imperatives for the preservation of joint function [1]. Septic arthritis predominantly manifests during childhood. The collective occurrence rate of septic arthritis in pediatric populations has been approximated within the range of 5.5 to 12 instances per 100,000 individuals [2]. Septic arthritis pertains to the invasion of a joint by a microorganism, often characterized by the presence of a swollen, highly sensitive joint, concomitant with systemic sepsis indicators such as fever and leukocytosis [3]. Inflammation of joints, or arthritis, manifests with diverse causative factors within the pediatric population.

*Staphylococcus aureus* stands as the predominant bacterial pathogen overall. Diverse age categories and deep-seated health issues are linked with unique etiology variables; these include conditions like rheumatoid arthritis, skin infections, liver disease, sickle cell anemia, and immunosuppressive medications. As an illustration, *Kingella kingae* is the most common gram-negative organism bacterium offender among the population under the age threshold of two to three years. *Neisseria gonorrhoea*, Group B Streptococcus, *Staphylococcus aureus*, and Gram-negative bacilli are frequently seen in newborns. Sickle cell disease (SCD) and salmonella creatures’ infestations are closely related. Long-term antimicrobial supplements increase the chance of individuals developing fungus infections. Joint infections caused by *Pseudomonas aeruginosa* are associated with injuries caused by puncture and the use of injectable medications. Among children, the hip joint emerges as the most frequently affected site in cases of joint involvement [4]. Specific subcategories of pediatric patients who are at an elevated risk encompass neonates, individuals with hemophilia who experience recurrent joint bleeding (hemarthroses), those with compromised immune systems (such as those with sickle cell anemia or human immunodeficiency virus infection), and individuals undergoing chemotherapy treatment [5]. Sickle cell disease is an autosomal-recessive genetic condition characterized by hemolytic anemia associated with aberrant hemoglobin and erythrocytes. Pediatric individuals who possess two copies of the sickle cell gene (hemoglobin SS) are notably predisposed to an elevated susceptibility to infections [6]. In pediatric patients, septic arthritis represents a recognized complication of SCD. In SCD patients with septic arthritis, the prevalent symptoms include pain and swelling in the affected joints, accompanied by fever often exceeding 38.2°C in a significant proportion of cases.

Elevated leukocyte counts, ranging from 7900 to 32,300/mm³, along with increased inflammatory markers such as a Westergren sedimentation rate greater than 24 mm/hour and C-reactive protein (CRP) levels exceeding 20 mg/L, are commonly observed. Positive joint aspirate cultures are a frequent diagnostic finding, with Staphylococcus and Gram-negative bacteria being the predominant causative agents, and a notable proportion of these infections occur in the hip joint [7]. The child refuses weight-bearing if the lower limb is involved [8]. The presence of a suppurative joint effusion linked to septic arthritis can potentially lead to pathological subluxation or dislocation if there is heightened intraarticular pressure for an extended duration [9]. The anatomically thin capsule may further contribute to pathological hip dislocation in infants [10]. Due to the proximity of the epiphyseal growth plate to the joint, the contiguous spread of a joint infection to any of the adjoining bones has the potential to result in diminished bone growth in pediatric patients because of epiphyseal plate distortion [11]. The presence of epiphyseal growth plate deformation may ultimately result in adverse outcomes, including joint destruction and impaired growth, underscoring the importance of timely intervention [12]. Fortunately, the current approach to managing septic arthritis in children primarily involves a combination of surgical interventions and antibiotic therapy to mitigate these potential complications [13]. Following surgical intervention for septic arthritis, postoperative care is advised to encompass specific physiotherapeutic measures. To mitigate the risk of joint contractures and preserve cartilage integrity, an initial period of immobilization and splinting is recommended. Additionally, an aggressive approach to physical therapy following soft-tissue surgery emphasizes its positive outcomes [14].

## Case presentation

Patient information 

The patient is a six-year-old female child who was brought to the outpatient department (OPD) by her mother. As reported by the mother, the patient's health was good until approximately six months ago. At that time, she began to manifest distressing symptoms, notably the gradual onset of persistent pain localized to the left hip region, not responding to analgesia. This discomfort extended in a radiating fashion to her left knee and was concomitant with episodes of high-grade intermittent fever, which exhibited amelioration following the administration of medication. The cumulative impact of these symptoms was a discernible challenge to the patient's ability to walk. Of noteworthy significance is the patient's established medical history, as she has been documented as a confirmed case of sickle cell anemia, characterized by an SS genetic pattern since she was a mere three months of age. In response to the aforementioned clinical presentation, the patient underwent a series of medical interventions. These included diagnostic hip aspiration and subsequent surgical management of septic hip arthritis by hip joint arthrotomy and soft tissue reconstruction. However, following the surgical procedure, she developed hip subluxation, which necessitated the application of distal femoral skeletal traction for therapeutic purposes. Upon removal of the skeletal traction, the patient continued to experience left hip stiffness, prompting the pursuit of soft tissue reconstruction and deformity correction procedures.

Clinical findings

The patient exhibited consciousness, cooperation, and orientation to time, place, and person during the initial evaluation at the department. Subsequently, after obtaining consent from both the patient and her mother, a physical examination was conducted with the patient in a supine position. The patient possessed an endomorphic body build and was found to have a recorded fever of 37.9 degrees Celsius. The patient's score on the numerical pain rating scale (NPRS) was 6/10 on rest and 8/10 on activity. The pain was aggravated during bed mobility and was relieved by rest and medications. Upon inspection, it was observed that the patient maintained her left hip in a posture characterized by flexion, abduction, and external rotation. Notably, surgical scars measuring approximately 4x2 cm were discernible on the lateral aspect of the left hip. Furthermore, an evident flexion deformity was identified in the left hip region. On palpation, a slight elevation in local temperature was noted, and the patient exhibited grade 2 tenderness in the left hip region. Additionally, her strength on manual muscle testing (MMT), which is mentioned in Table 1, revealed reduced strength in the lower limbs.

**Table 1:** Bilateral manual muscle testing of lower limbs

Muscles	Right	Left
Hip flexors	Grade 4	Grade 3
Hip extensors	Grade 4	Grade 3
Hip abductors	Grade 5	Grade 3
Hip adductors	Grade 4	Grade 3
Hip external rotators	Grade 4	Grade 3
Hip internal rotators	Grade 4	Grade 3
Knee flexors	Grade 5	Grade 4
Knee extensors	Grade 5	Grade 4
Ankle plantar flexors	Grade 5	Grade 5
Ankle dorsiflexors	Grade 5	Grade 5
Ankle evertors	Grade 5	Grade 5
Ankle invertors	Grade 5	Grade 5

The assessment revealed a painful and restricted range of motion (ROM) in the hip joint shown in Table 2, along with the presence of tightness in both the rectus femoris and tendoachilles.

**Table 2:** Range of motion of hip, knee, and ankle joints of both lower limbs

ROM	Right	Left
Hip flexion	0-90°	0-40°
Hip extension	0-30°	0-10°
Knee flexion	0-130°	0-50°
Knee extension	130°-0	50°-0
Ankle plantarflexion	0-50°	0-35°
Ankle dorsiflexion	0-20°	0-10°

Diagnostic assessment

**Figure 1:**
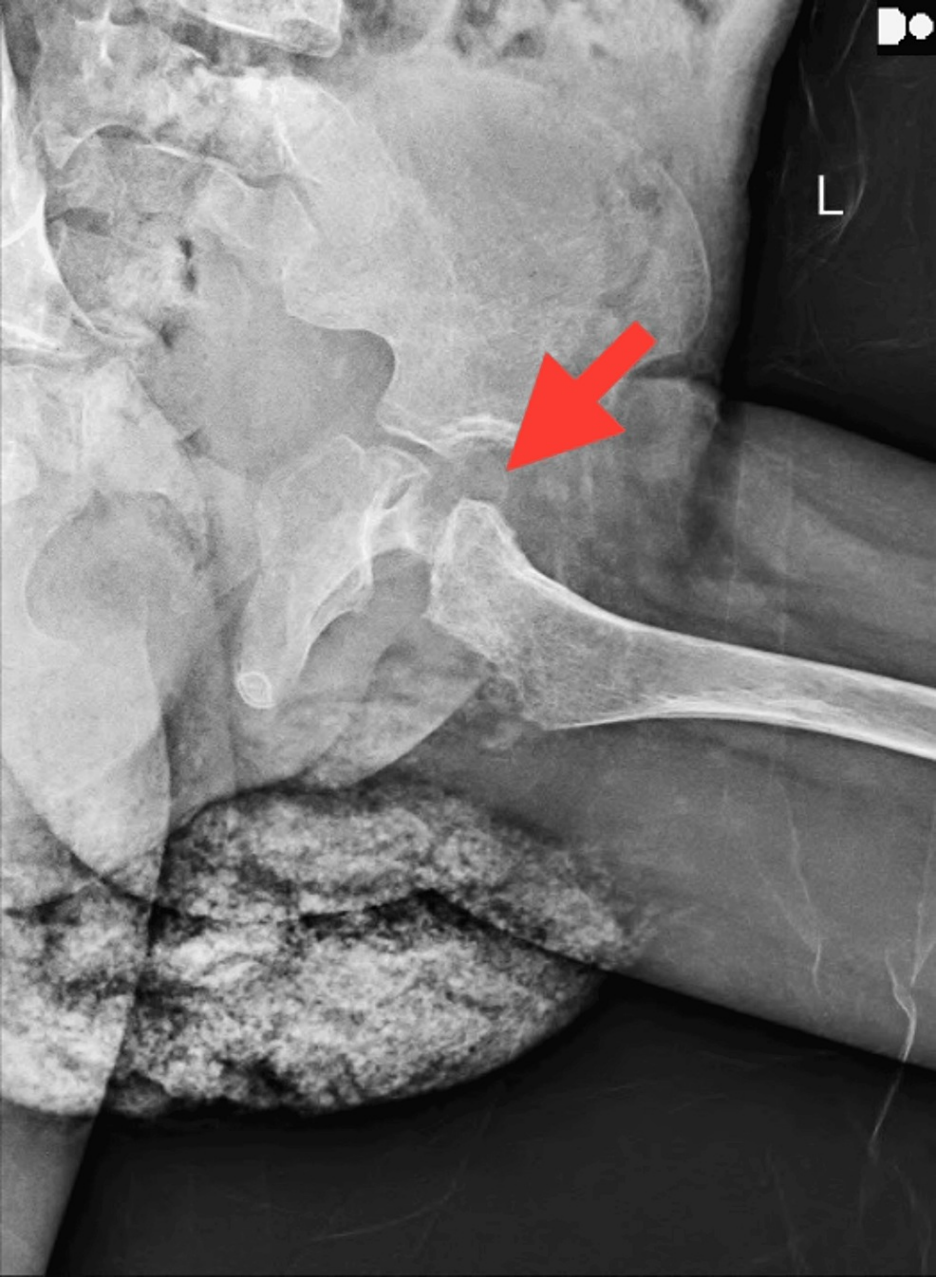
Radiographic imaging of left hip joint

**Figure 2:**
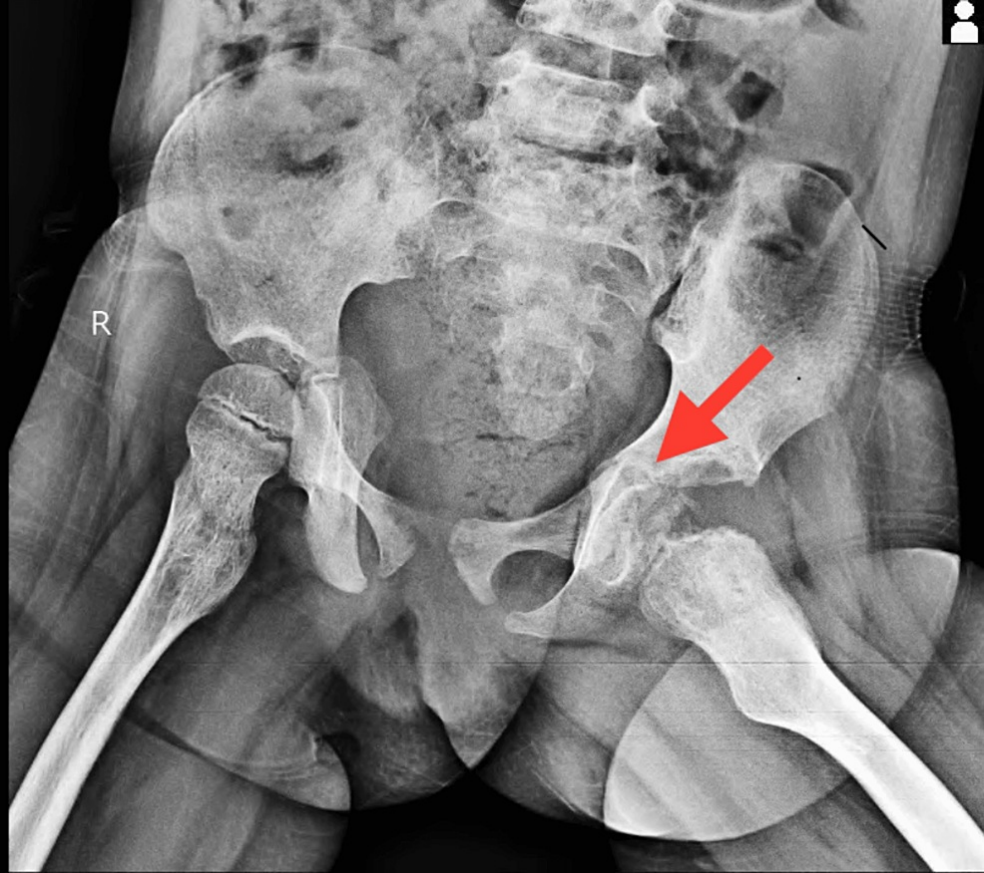
Radiographic imaging of bilateral hip joints (AP view) AP: anteroposterior

**Figure 3:**
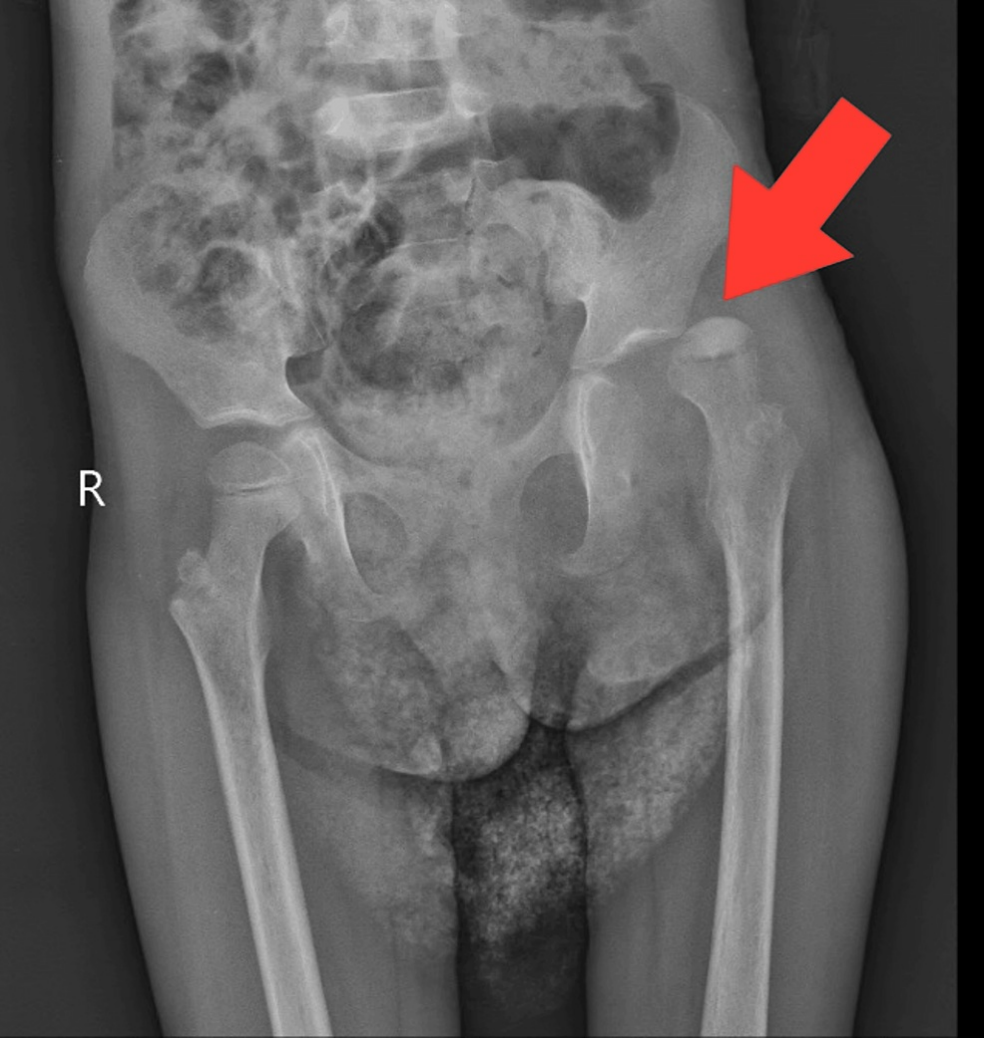
Radiographic imaging of bilateral hip joint

Hematological investigations in the patient's evaluation revealed a reduction in hemoglobin levels, an elevated total white blood cell count, and microcytic hypochromic red blood cells demonstrating anisopoikilocytosis, including the presence of pencil cells, sickled red blood cells, occasional nucleated red blood cells, and fragmented red blood cells. The culture reports from the patient's pus swab demonstrated the proliferation of *Pseudomonas aeruginosa*. Analysis of radiographic images from X-ray scans disclosed findings of sclerotic alterations as shown in Figure 1, bowing of the femoral shaft, rotational changes in the femoral shaft, soft tissue swelling around the joint and within the joint capsule, repositioning of the fat pad, decreased joint space as shown in Figure 2, attributed to localized edema and effusion, as well as evident epiphyseal plate deformities and hip subluxation shown in Figure 3.

**Figure 4:**
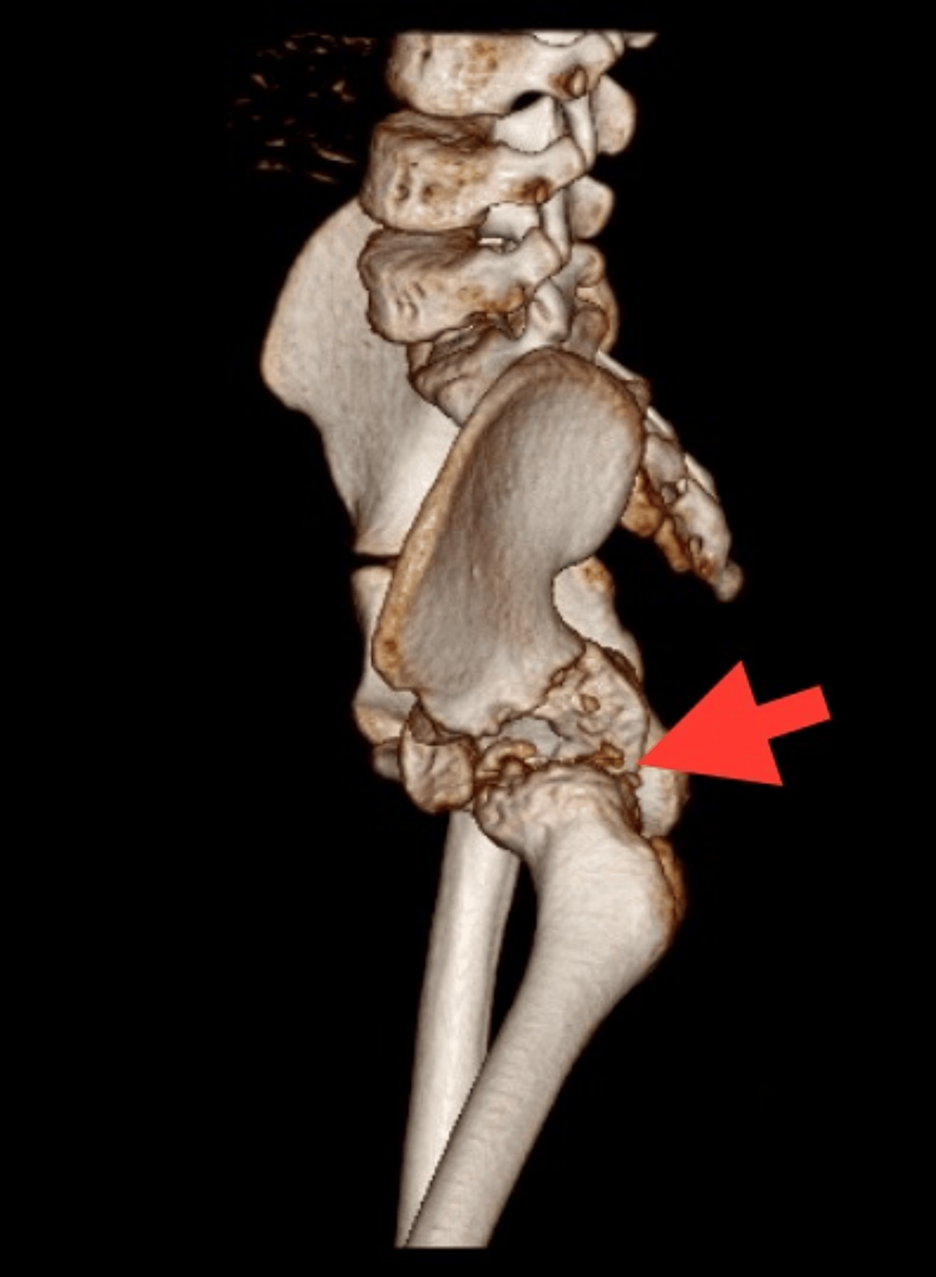
3D CT scan of the lateral view of the left hip joint 3D: three dimensional; CT: computed tomography

**Figure 5:**
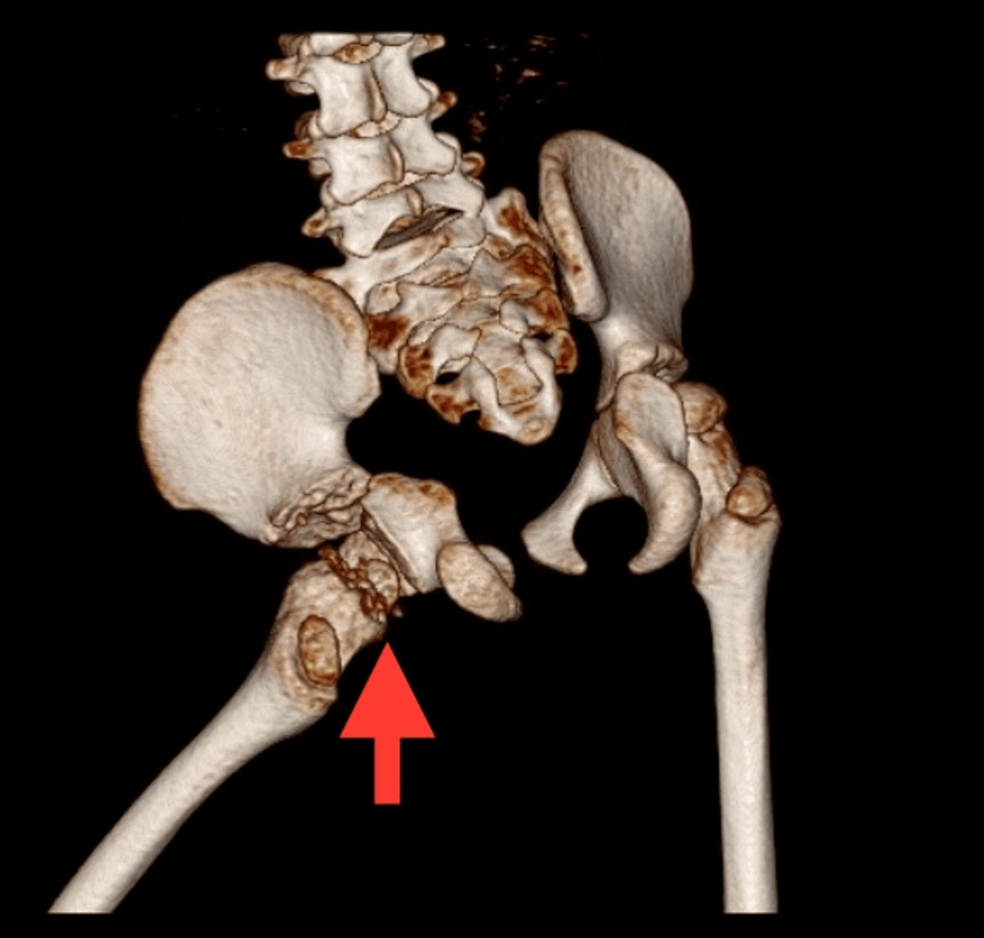
3D CT scan of the posterior view of the left hip joint 3D: three dimensional; CT: computed tomography

Computed tomography (CT) scans exhibited signs of erosion in bone and cartilage, along with synovial thickening, as shown in Figures 4-5.

Diagnosis: septic arthritis-induced hip subluxation and epiphyseal plate deformation in a patient with sickle cell anemia

Therapeutic Interventions

Table 3 depicts the phase-wise intervention planned for the patient's treatment.

**Table 3:** Stage-wise progression of the rehabilitation protocol ROM: range of motion

Rehabilitation phases	Goals	Dosage and interventions
Phase 1: Early postoperative phase (0-2 weeks)	Education of the patient	The patient was provided with information on potential dangers connected with mitigating their dependence, the advantages of physiotherapy, the importance it has in strengthening their ability to participate in behaviors such as strolling and bending over, as well as the significance that it offers for boosting their overall wellness.
	To retain muscle strength	The regimen includes isometric contractions involving the quadriceps and hamstrings, comprising 10 repetitions with a five-second sustained contraction within a single set. Additionally, upper-limb strengthening exercises have commenced, utilizing a half-liter water bottle for resistance.
	To improve ROM	To achieve a modest degree of flexion at the knees, an unrestrained passive motion was commenced, as shown in Figure 5. Ten repetitions of straight leg raising for the unaffected extremity were performed to maintain the range of motion.
	To avoid unforeseen problems	Relaxed passive ankle toe movements for 10 repetitions for one set were performed as depicted in Figure 6.
	To ensure sufficient airflow	Thoracic expansion exercises for 10 reps for a single set were performed as shown in Figure 7.
Phase 2: Intermediate phase (4-6 weeks)	To improve muscle strength	Advancement to resistance exercises involving both open and closed kinematic chain movements.
	Joint mobility	More than half of the ROM of the hip and knee is achieved.
Phase 3: Return to activity phase (8 weeks and beyond)	Muscle performance and joint mobility	Full active resisted range of motion exercises were continued, and progression was made for open and closed kinematic chain exercises, and there was a complete return of functional activity by this time.

**Figure 6:**
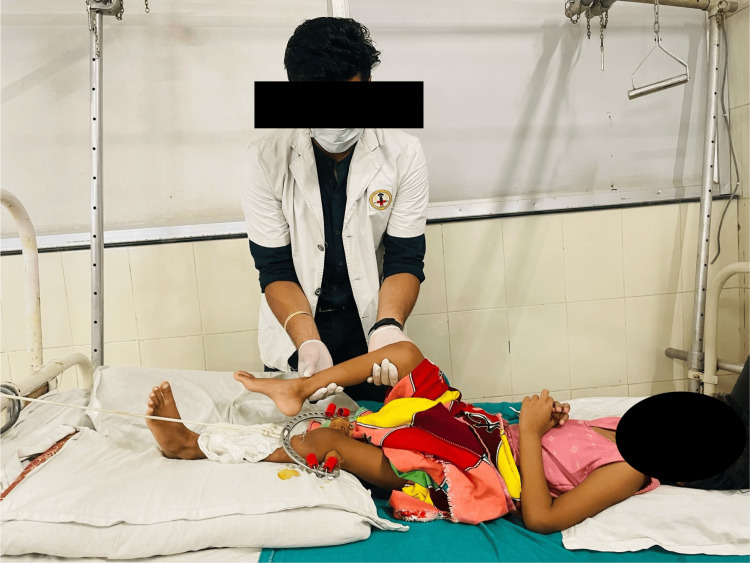
Passive ROM exercises for unaffected lower extremities ROM: range of motion

**Figure 7:**
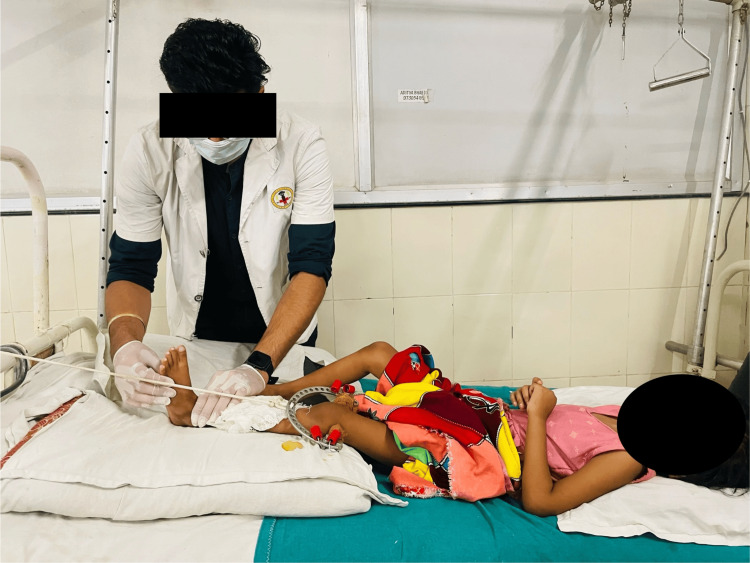
Relaxed passive ankle toe movements for the left lower limb

**Figure 8:**
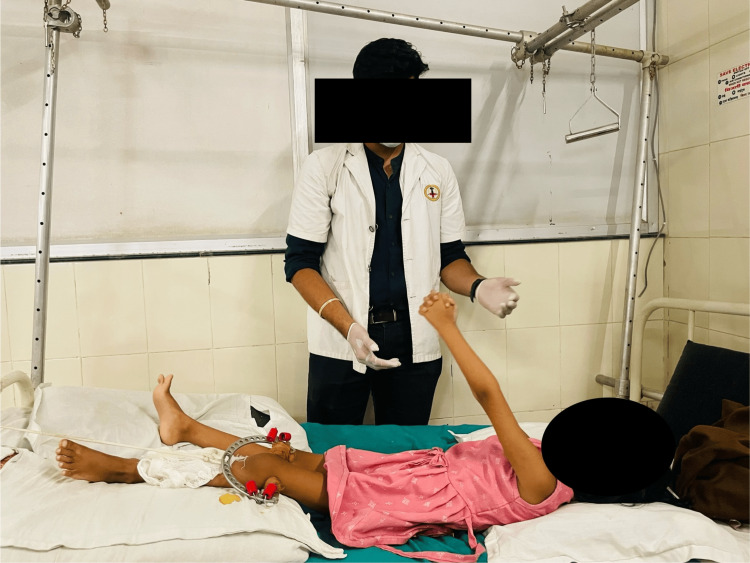
Thoracic expansion exercises for improving ventilation

Figures 6-8 showcase the exercises that were a part of the rehabilitation protocol. 

Follow-up and outcome of interventions

After being discharged, the six-year-old female child, previously diagnosed with septic arthritis in the hip, participated in an all-encompassing rehabilitation program under the supervision of a musculoskeletal physiotherapist. The initial evaluation revealed that the patient encountered hip discomfort, achieving a pain rating of 6/10 on the NPRS while at rest and 8/10 during physical activity. Her hip's ROM was both constrained and painful, accompanied by a manual muscle testing score of 3/5. The Hip Disability and Osteoarthritis Outcome Score-12 (HOOS-12) was determined to be 44. Throughout the 12-week rehabilitation program, notable advancements were observed. Upon concluding the treatment, the patient conveyed the absence of pain and enhanced ease in executing everyday activities. Her functional ROM exhibited significant improvement. To assess the outcomes of the rehabilitation regimen, the antecedent and subsequent parameters, encompassing pain levels, symptom intensity, activities related to daily living, functionality in sports and recreational pursuits, and the quality of life pertaining to the hip, were evaluated employing the Hip Injury and Osteoarthritis Outcome Score (HOOS) shown in Table 4.

**Table 4:** Pre- and post-rehabilitation ROM and MMT of the right lower limb and HOOS score NPRS: numerical pain rating scale; ROM: range of motion; MMT: manual muscle testing; HOOS: Hip Injury and Osteoarthritis Outcome Score

	Pre-rehabilitation	Post-rehabilitation
NPRS	On rest: 6/10; on activity: 8/10	On rest : 2/10; on activity: 3/10
ROM of left lower extremity		
Hip flexion	0-40°	0-90°
Hip extension	0-10°	0-30°
Knee flexion	0-50°	0-100°
Knee extension	50°-0	100°-0
MMT of left lower extremity		
Hip flexors	Grade 3	Grade 4
Hip extensors	Grade 3	Grade 4
Knee flexors	Grade 4	Grade 5
Knee extensors	Grade 4	Grade 5
HOOS score	42	80

This appraisal employs a five-dimensional scale, featuring scores that span from 0 (indicating the absence of complications) to four (indicating the presence of extreme challenges). The ultimate score is translated to a 0-100 scale, with 0 representing severe complexities and 100 denoting the absence of difficulties.

## Discussion

Septic arthritis in pediatric patients invariably constitutes a medical emergency, with a notable predilection for impacting the articulations of the lower limbs, particularly the hip and knee [15]. Prompt recognition and management of septic arthritis affecting the hip are of paramount significance. A delayed diagnosis can lead to permanent destruction of the hip joint. Prolonged joint effusion with sustained intra-articular pressure over an extended period, be it days or weeks, may give rise to hip subluxation or, in severe cases, dislocation. Moreover, the persistence of purulent effusion in a joint for more than four days will invariably lead to irreversible harm to both the joint cartilage and the epiphyseal growth plates [16]. Infection parameters may not always manifest clearly. The pediatric patient may exhibit either a severe illness, akin to cases of septicemia, necessitating immediate life-saving interventions, or display subtle signs of infection, such as a rising body temperature. Typical clinical manifestations and indications encompass the child's overall poor condition, severe pain upon joint movement, the loss of weight-bearing capacity, limping, and a septic appearance. Diagnosing the condition in infants can be particularly challenging since they may not exhibit the typical fever associated with sepsis. A meticulous clinical examination of the patient plays a pivotal role in ensuring successful treatment. In childhood, the primary etiology of infection is hematogenous transmission, culminating in bacterial colonization of the joint. Distinguishing between septic arthritis and transient synovitis of the hip in pediatric cases can prove to be intricate.

In 1999, Kocher et al. introduced four highly reliable positive predictive indicators (99.6%) for septic arthritis, which include: fever (temperature ≥38.5°C), inability to bear weight, an elevated white blood cell count >12.0 x 10^^9^ cells/L, and an erythrocyte sedimentation rate (ESR) ≥40 mm/hour. Later, CRP was added as a fifth predictor (CRP ≥20 mg/l). Conversely, transient synovitis of the hip stands as the most prevalent cause of hip pain in school-age children, whereas septic arthritis of the hip is a rare occurrence. The conclusion drawn is that favorable outcomes are primarily associated with recent dislocations, emphasizing the significance of prompt arthrotomy and immobilization [17]. Due to the aforementioned articular changes, physical rehabilitation plays a pivotal role in preventing joint inflammation and muscle wasting and restoring patients to a high level of daily functioning. An initial patient assessment may involve employing the HOOS score, which assesses various aspects, including symptoms, stiffness, pain, daily activities, sports and recreational engagement, and overall quality of life. To mitigate subsequent joint damage and manage patient discomfort during joint mobilization, the affected joint is initially immobilized. Following surgical intervention, the patient is immobilized and splinted as an early precaution against joint contractures, and active or passive range of motion exercises are introduced to preserve cartilage integrity post-surgery. Obtaining a comprehensive patient history is crucial, with pediatric orthopedics typically relying on parental input for information. The extent of joint recovery hinges on the degree of joint damage, which can be radiologically assessed using the Bennett and Namnyak grading system. Pain assessment involves a 0-10 numeric written scale administered at the outset of physical therapy, allowing the patient to detail the pain's characteristics, location within the hip region, and how it impacts daily activities and rest. Physiotherapy regimens are tailored according to the severity of joint damage, encompassing multidirectional stretching in cardinal planes, maintained at the end range for 10 to 20 seconds, or as tolerated by the patient. Active-assistive range of motion exercises for the lower extremity and self-distraction of the affected hip in a long-axis direction are included. For patients with extended periods of immobilization, exercises involving weight shifts, heel lifts, lateral movements, and preparations for walking between parallel bars can be introduced. Patients receive instructions for a home exercise program that incorporates both active and passive hip stretching, with an emphasis on hip extension exercises, performed three times daily for five minutes each. Contract-relax stretching exercises targeting the iliopsoas and hamstrings are also recommended. Manual therapy techniques and specific directional glides are integrated into the treatment plan, along with an early repetitive passive range of motion exercises aimed at potentially regenerating the articular surface. In cases where joint resistance to mobilization persists, manipulation techniques under anesthesia may be employed, followed by a resumption of activity. The focus lies on enhancing the joint ROM throughout the rehabilitation process.

This case highlights the severity of septic arthritis in a pediatric patient, particularly in the hip and knee. Early diagnosis and intervention are crucial. A six-year-old patient with sickle cell anemia experienced persistent hip pain, leading to surgery and hip subluxation. Clinical findings included limited mobility, weakness, and radiographic abnormalities. After comprehensive rehabilitation, significant improvements in pain, mobility, and function were observed. Early diagnosis and timely intervention play a vital role in mitigating septic arthritis's impact on joint function and overall outcomes. In the context of managing SCD sequela, it is imperative to emphasize the importance of parental education. Parents of children affected by SCD should be well-informed about the potential risk of developing septic arthritis. By providing educational resources and guidance, healthcare professionals can empower parents to recognize early warning signs and red flags indicative of septic arthritis. This heightened awareness is vital for facilitating timely medical intervention, thereby mitigating the risk of diagnostic delays and ensuring prompt and effective management.

## Conclusions

This case report highlights the paramount significance of expeditious diagnosis and all-encompassing restorative measures when addressing septic arthritis in the pediatric patient cohort, particularly those afflicted by underlying maladies such as sickle cell anemia. The primary lessons to glean from this case report can be succinctly encapsulated as follows: septic arthritis in the pediatric demographic, with a pronounced predilection for substantial joint involvement, notably the hip and knee, necessitates immediate attention. This illustrative case underscores the profound importance of recognizing and diagnosing the condition with swiftness, as this expedience is the linchpin for averting permanent articular devastation. Children harboring underlying medical conditions, exemplified by the likes of sickle cell anemia, manifest a heightened predisposition to septic arthritis. Thus, healthcare practitioners are enjoined to maintain a vigilant stance in the surveillance and diagnostic evaluation of joint-related maladies within these susceptible subpopulations. Effective management of septic arthritis in the pediatric realm necessitates a multidisciplinary approach encompassing orthopedic surgical intervention, judicious antibiotic therapy, and meticulous physiotherapeutic modalities. The case lucidly illustrates the imperative of an impeccably orchestrated treatment schema that maximizes the prospects of optimal clinical outcomes. Tailored rehabilitation, calibrated in accordance with the extent of joint impairment, emerges as a prerequisite for the preservation of articular functionality, mitigation of complications, and the reinstatement of pediatric patients to a zenith of diurnal operation. Systematic assessment of pain indices, range of motion parameters, and various functional metrics is of the essence in effecting meticulous tracking of progress and the judicious adaptation of rehabilitation methodologies to concomitant clinical exigencies. This case underscores the dire consequences of septic arthritis in pediatric patients, especially with underlying conditions. Swift diagnosis, timely treatment, and comprehensive rehabilitation are vital for improving pain relief and mobility. It highlights the effectiveness of a coordinated healthcare team for vulnerable patients.
